# Vascular endothelial growth factor receptor-1 (VEGFR-1) expression in human corneal fibroblast decreased with age

**Published:** 2009-09-29

**Authors:** Alexandre Berthaut, Pezhman Mirshahi, Nadia Benabbou, Dalel Azzazene, Camille Bordu, Amu Therwath, Jean-marc Legeais, Massoud Mirshahi

**Affiliations:** 1 INSERM UMRS 872, E18, Faculté de Médecine Paris VI, Paris, France; 2 Laboratoire de Biotechnologie et Oeil, Université Paris 5, Paris, France

## Abstract

**Purpose:**

Mechanisms by which fibroblast networks between stromal lamellae are laid in the corneal stroma are far from clear. We have investigated the role of vascular endothelial growth factor receptors (VEGFRs) by in vitro studies in the human corneal network formation obtained from donors whose ages ranged from 19 to 89 years.

**Methods:**

Corneal fibroblasts were prepared from cornea donations. The functional properties of these cells to form networks were analyzed using a semi solid matrix (substratum) of Matrigel™. The presence of VEGF receptor-1 (VEGFR-1) and the functionality in these fibroblasts were investigated using immunofluorescence, molecular analysis (gene microarray, reverse transcription polymerase chain reaction [RT–PCR] and VEGFR siRNA transfections), and cell culture.

**Results:**

Corneal fibroblasts from 61 donors were classified into two groups according to whether they formed (82%) a reticulum on Matrigel™ or not (18%). By RT–PCR and immunofluorescence analysis, we showed that corneal fibroblasts expressed VEGFR-1 (mRNA and protein). Further, cell culture analysis revealed that only the network (reticulum) forming corneal fibroblast expressed VEGFR-1 in contrast to non network-forming fibroblasts. Use of inhibitors such as *VEGFR-1* siRNA transfection or neutralizing antibody (Avastin™) indicated that VEGFR-1 was essential to the formation of the corneal network in vitro.

**Conclusions:**

The cell reticulum formation seemed to be directly related to the expression of *VEGFR-1* in the corneal fibroblast, and this expression decreased with age. The decrease in *VEGFR-1* expression is probably related to the diminution of autocrine functions, which may alter the overall tissular homeostasis. This may culminate in the gradual development of poor vision, which is observed in certain pathologies and in aging individuals.

## Introduction

The cornea is a highly differentiated tissue, rich in extracellular matrix (ECM) synthesized by its cell constituents. The corneal matrix is characterized by a well-defined supramolecular structure that ensures the dual function of the cornea, transparency as well as inner-eye tissue protection. ECM disorders have been associated with various eye diseases such as corneal macular dystrophy and Marfan’s syndrome.

The human corneal stroma is a stacking of collagenous lamellae. They are colonized with keratocytes (quiescent cells) or following wound healing, with corneal fibroblasts or myofibroblasts (activated cells derived from keratocytes) [1-3]. Several investigations have shown that the arrangement of keratocytes within the corneal stroma is an essential mechanism in the maintenance of corneal transparency. Scanning electron microscopy has revealed a network of flat cells with many small dendritic bifurcations in the rat cornea [4]. Studies on rabbit and human cornea using immunocytochemistry [5,6] and electron microscopy [7-9] have described the existence of gap junctions between keratocytes. Flat sections of the cornea show that the fibroblasts have long branching processes or digitations, which extend in several directions from the cell body and establish contacts with similar processes of other cells in the vicinity. According to several recent reports [10], these cells appear to form a syncytium-like structure. This however contradicts earlier observations by others who used electron micrographs, which showed the presence of almost a 200 Å wide intercellular space separating these cells [11]. The structural morphology of intercellular connections of the corneal fibroblast was shown in flat sections of the corneal stroma. It reveals the existence of a functional circuit of a communication network between keratocytes [10]. However, knowledge of the detailed mechanisms involved in the corneal fibroblast network formation still eludes us. We have been interested in the process of corneal fibroblast reticulation for a few years now and have tried to further our understanding through biochemical and cell biology studies. The fibulins, a family of the ECM proteins, were characterized by our group recently for their implication in the reticulation of organ shape and stromal cell motility [1].

Due to its avascularity, the cornea has not only served as the principal in vivo model system for studying angiogenesis but also as a convenient model for vascular endothelial growth factor (VEGF) and vascular endothelial growth factor receptor (VEGFR) signaling pathways in several physiopathological disorders [12].

VEGF encompasses a family of structurally related proteins that include PlGF (placental-derived growth factor), VEGF-A, VEGF-B, VEGF-C, VEGF-D, and VEGF-E. The VEGF receptor family in mammals contains three members, VEGFR-1 (fms-like tyrosine kinase-1 or flt-1), VEGFR-2 (kinase insert domain containing receptor/fetal liver kinase-1 or KDR/flk1), and VEGFR-3 (fms-like tyrosine kinase-4 or flt-4) in addition to a co-receptor, NRP1 (neuropilin). These factors directly participate in the genesis of blood capillaries and lymphatic vessels [13-18].

It has been shown that corneal wound healing requires the action of several angiogenic factors that include VEGF and b-FGF (basic fibroblast growth factor) [19-21]. The circulating soluble receptors, VEGFR-1 and VEGFR-3, play a crucial role in the avascularity of the cornea. It acts by binding free VEGF thereby making it unavailable for binding to receptors on cell membranes [22,23].

Knowledge regarding the role of VEGF, the mechanisms underlying corneal stromal cell integrity, and the complex inter-cellular network formation is still very scant. Our laboratory has been interested in understanding the complexity involved in corneal integrity and has undertaken several lines of research aimed at it for the past few years. In the present study, we focus on the role of VEGFR-1 in the human corneal stromal fibroblast network organization in vitro.

## Methods

### Isolation and primary culture of corneal stromal cells

Human corneas were obtained from the “Banque Française des yeux” after informed consent of the donors in agreement with the revised rules of the Helsinki Protocol. Pieces of cornea 2–3 mm^2^ in size were gently scratched to remove epithelial cells. The corneal pieces were seeded on 24 well plates containing Dulbecco’s Modified Eagle’s Medium:F12 (DMEM/F12; PAA, Mureaux, France) supplemented with 10% fetal calf serum (FCS), penicillin (100 U/ml), and streptomycin (100 µg/ml; PAA) and incubated at 37 °C in a humidified atmosphere containing 5% CO_2_. Non-adherent cells were removed and discarded after three days and the culture dishes were washed with 1× phosphate buffer saline (1× PBS; PAA). After an additional week in culture, some cells began to spread out of the corneal explants. These cells were primary corneal stromal cells and were detached with trypsin treatment (PAA) for 15 min. The cells were centrifuged, and the pellet was washed, resuspended, and seeded in the same complete medium for incubation for two weeks in six-well plates (4×10^4^ cells/cm^2^) in a humidified atmosphere containing 5% CO_2_ at 37 °C. Samples were taken and used for experiments from the first passage onwards through the sixth.

### Wound healing test

Motility and adhesion were analyzed on cells grown to 80% confluency in 24 well culture dishes coated with “Attachment Factor” (PAA). Then, four scars/wounds (two vertical and two horizontal) were made using a blunt glass tip. The dishes were then incubated in a DMEM/F12 medium containing only 2% FCS, and triplicate dishes were assigned for (a) the control, (b) with Avastin (10^−7 ^M), (c) with VEGF-A (100 ng/ml), and (d) with VEGF-C (100 ng/ml). In the presence of 2% FCS, cell proliferation did not occur, but cell viability was unaffected. The number of cells that migrated into the wound track were counted during incubation at intervals of 9 h, 18 h, 24 h, 48 h, and 72 h and the mean number of cells computed (n=4).

### Immunocytochemistry

One million cells were cultured for 24 h in glass chamber slides (Labtek®; Nunc, Naperville, IL) in complete DMEM/F12. Cells were washed with a serum-free medium and fixed for 15 min with 3% paraformaldehyde in 1× PBS. After three washes with PBS, cells were further incubated for 20 min with 1% (w/v) bovine serum albumin (BSA; Sigma-Aldrich, St Louis, MO) to avoid non-specific binding. Cells were exposed to VEGFR-1 antibody for 1 h at 4 °C. After three washes with PBS, cells were treated with anti-goat secondary biotinylated antibody for 1 h at 4 °C (1:100 dilution). Cells were washed twice in PBS-BSA and exposed to Streptavidin-Fluorescein (Sigma-Aldrich) for 45 min at 4 °C. The antibody fluorescence was observed on slides using a Nikon fluorescence microscope (Nikon, Paris, France).

### Matrigel™ assays

The cell motility and cell-cell contacts were assessed on Matrigel™, a natural murine fibrosarcoma extracellular matrix (BD Biosciences, San-Jose, CA). Matrigel™ provides a physiologically relevant environment for studies of cell morphology, migration, and invasion as cultures grown on top of it presents a two-dimensional situation for cellular network formation. A 96 well microplate was coated with 35 µl of Matrigel™. After 1 h at 37 °C, 10^4^ cells in 200 µl of DMEM:F12 and VEGF (100 ng/ml; PromoCell GmbH, Heidelberg, Germany) were seeded into each well. Culture cells were observed using an inverted microscope, and cell-cell contacts (intercellular junctions) as well as tubular reticulum formation were counted at 3 h intervals and the results plotted as a histogram.

### Analysis by gene array

The gene expression patterns of corneal primary culture cells and human corneal fibroblasts immortalized by SV40 T antigen transfection [24] were analyzed and compared by a two-color topic-defined microarray (PIQOR Microarray, Miltenyi Biotec GmbH, Bergish Galdbach, Germany) as described below. Briefly, 5×10^6^ cells each (primary corneal culture cells as well as immortalized corneal fibroblasts) were lysed in the Trizol® reagent (Invitrogen, Carlsbad, CA) and shipped to Miltenyi Biotec GmbH in dry ice. Total RNA was extracted, reverse transcribed, amplified, and fluorescence coupled with Cy3 (primary culture cells) or Cy5 (immortalized corneal fibroblasts) and then hybridized on PIQOR^TM^ microarrays. The capture of images and signal quantification were performed by ScanArrayLite (Packard Bioscience, Billerica, MA) and ImaGene software Version 4.1 (BioDiscovery, Los Angeles, CA). Local background was subtracted to obtain net signal intensity, and the Cy5/Cy3 ratios were calculated. The results were analyzed by analysis of variance (ANOVA) test, and the mean ratios of quadruplicate spots were normalized using locally weighted linear regression by LOWESS (locally weighted scatter plot smoothing) normalization method. Only genes displaying net signal intensity twofold higher than the mean background were considered significant and used for further analysis. The products of microarray consisted of genes related to cytokines, interleukins, and growth factors.

### Transfection protocols

Primary fibroblast cultures were prepared as described above and used when they attained 80% confluence. DMEM:F12 complete medium was removed from each six-well plate containing adherent cells and washed once with PBS. Cells were detached by Accutase® (PAA) and resuspended to give a concentration of 10^5^ cells per 100 µl.

In the transfection assay, the siRNA-couple (Qiagen, Hilden, Germany) for each mRNA was tested along with a control sample using scrambled siRNA to determine changes in cell phenotype, if any. A second control was also done using only the medium without siRNA.

To ascertain the best suited protocol for transfection of our cells, we have tried three different methods.

#### Amaxa® transfection protocol

This is essentially an electroporation protocol consisting of the following: One million cells with 100 µl of Nucleofactor™ solution (Amaxa; Lonza, Cologne, Germany) and 2 µg siRNA (for each sample) was mixed in Eppendorf tubes and transferred to Amaxa certified cuvettes at 37 °C. The manipulations were quick and lasted less then 15 min to avoid cell mortality and thus adversely affect efficiency of gene transfer. At the end of electroporation, 500 µl of the pre-warmed DMEM:F12 containing serum at 37 °C was added to each cuvette. The cells were seeded and incubated for 24 h in a humid atmosphere at 37 °C with 5% CO_2_.

#### Hiperfect® Qiagen protocol

The siRNA-couple was diluted (37.5 ng/100 μl) in serum free-medium. Three microliters of HiPerfect transfection reagent (Qiagen) was added to the diluted siRNA and mixed by vortexing. The samples were incubated for 10 min at room temperature to allow formation of transfection complexes. These complexes were added onto 10^5^ cells per 100 µl and incubated for 6 h at room temperature after which 300 µl of the medium containing 10% FCS was added in each well and incubated further for 24 h in a humid atmosphere at 37 °C with 5% CO_2_.

#### Nanofectin® transfection protocol

The stock solutions of siRNA and Nanofectin siRNA were used at room temperature. Half a microgram of siRNA (i.e. 15 pmol) in 30 µl of serum free-medium was added to each well. Two and a half microliters of Nanofectin (PAA) was added to siRNA in 40 μl of serum free-medium in a second series of wells. The two series of wells were incubated separately for 20 min at room temperature after which the solutions from the first and second series were pooled together giving a final volume of 70 μl for the siRNA nanocomplex mixture. This solution was added drop-wise to 10^5^ cells in 500 µl of medium with or without FCS. The incubation continued at room temperature for 6 h. Cells were pooled and centrifuged. The washed pellet was resuspended for another 24 h incubation in a humid atmosphere at 37 °C with 5% CO_2_. At the end of the incubation and for each transfection protocol tested, a viability test with Trypan Bleu® (Sigma-Aldrich) was performed and the number of living and dead cells was determined.

### PCR procedure

Total RNA was extracted from primary corneal fibroblasts using Nucleospin RNA-II kit (Macherey-Nagel, Hoerdt, France). Cells (5×10^6^) were lysed at 0 °C, and RNA and DNA were selected on the silicate membrane. DNA was degraded directly on the silicate membrane using DNase I for 15 min at room temperature. Following several washes, total RNA was eluted with RNase free water and the integrity of RNA verified on a 2% agarose gel (Sigma-Adrich) in 0.45% ethidium bromide and quantified by spectrometry at the wavelength of 260–280 nm. One microgram of total RNA was used for reverse transcription to create cDNA according to the standard protocol. We used the following synthetic primers (Eurobio, Paris, France): VEGFR-1 sense 5′-CAC CAA GAG CGA CGT GTG-3′, antisense 5′-TTT TGG GTC TCT GTG CCA G-3′ (196 bp) and β2microglobulin sense 5′-CCA GCA GAG AAT GGA AAG TC-3′, antisense 5′-GAT GCT GCT TAC ATG TCT CG-3′.

The reaction mixture consisted of 15 ng of cDNA and 2.5 units Taq polymerase (GibCo BRL, Paisley, UK), and the amplification was performed according to the protocol recommended by GibCo BRL. The melting temperature (T_m_) for VEGFR-1 and for β2microglobulin was 60 °C. Polymerase chain reaction (PCR) products were analyzed on 2% agarose gel electrophoresis in 0.45% ethidium bromide. The PCR cycle number was normalized to the amount of human β2-microglobulin cDNA product.

### Statistical analysis

The Fischer test: statistical analysis was performed using the modified FISHER test. The data was expressed as standard  error of the mean (SEM). A p value less than 0.05 was considered statistically significant.

## Results

### Network formation by corneal fibroblasts on Matrigel™

**Figure 1:**
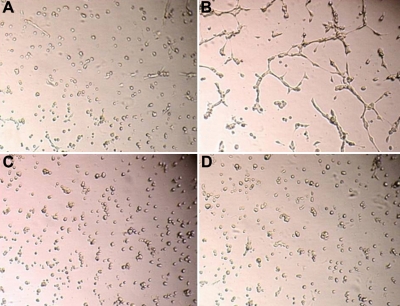
Network formation by corneal fibroblasts on Matrigel. Type-1(**A**) and type-2 (**C**) corneal fibroblasts were seeded on matrigel and photographed before incubation, representing time 0.  The same plates were photographed after 2 h of incubation. **B** (type-1 corneal fibroblast) shows cell elongation and beginning of network formation whereas **D** (type-2 fibroblast) shows no network formation, and resembles the unincubated controls at time 0.

Network formation of corneal fibroblasts is presented in Figure 1. Primary culture cells from 62 donors were seeded on Matrigel™. Corneal cells from 51 donors (82%) organized into tubules while 11 donors (18%) remained dispersed (unorganized). These results suggest that the corneal fibroblasts from donors belong to two distinct categories and can be classified into type 1 and type 2 according to whether they form (Figure 1A,B) or do not form (Figure 1C,D) a network on Matrigel™. Normally, after 2 h, cell network formation starts, and cell-cell interaction lead to the formation of a cell network as described in “Reticulogenesis” [25].

### Gene array analysis

Table 1 indicates that corneal fibroblasts in primary culture (n=4) or the immortalized cell line (n=1) express *VEGF-A*, *VEGF-C*, *VEGFR-1*, and *NRP1* mRNA but not *VEGFR-2*, *VEGFR-3*, and *VEGF-D* mRNA. These results were also confirmed by reverse transcription polymerase chain reaction (RT–PCR) analysis using specific primers.

**Table 1:** Gene array analysis.

**Gene name**	**UniGene code**	**Gene array analysis**
VEGF		
*VEGFA*	Hs.73793	positive
*VEGFB*	Hs.78781	positive
*VEGFC*	Hs.435215	not tested
*VEGFD*	Hs.113922	positive
*VEGFE*		not tested
*PlGF*	Hs.252820	negative
VEGF receptors		
*VEGFR-1*	Hs.654360	positive
*VEGFR-2*	Hs.479756	negative
*VEGFR-3*	Hs.646917	negative
VEGF co-receptors		
*NRP1*	Hs.131704	positive
*NRP2*	Hs.471200	not tested

*VEGF*, *VEGFR*s and *NRP* mRNA expression by human corneal fibroblast cells. Column 1indicates the names of different genes that were tested by gene array. Column 2 indicates their corresponding UniGene code. Column 3 shows whether the gene was positive or negative for mRNA expression.

### Demonstration of VEGFR-1 protein in corneal fibroblasts

**Figure 2:**
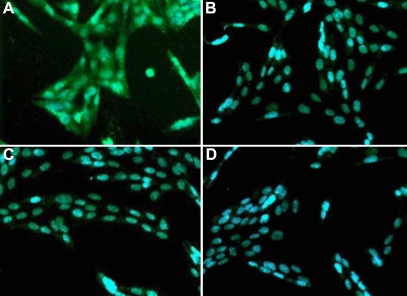
Demonstration of VEGFR1 protein in corneal fibroblasts by immunofluorescence. The presence of VEGFR1 was investigated using antibodies specific to VEGFR1 and VEGFR2. **A**: Significant levels of VEGFR1-specific fluorescence in type 1 donor fibroblasts. VEGFR1 immunoreactivity was undetectable in type 2 donors cells (**C**).  **B** (type -1) and **D** (type-2) represent cells treated with anti-VEGFR2 antibodies, providing negative controls.

The presence of VEGFR-1 was determined by immunofluorescence. A significant level of VEGFR-1 was expressed by type 1 donor fibroblasts (Figure 2A) whereas the VEGFR-1 immunoreactivity was low in corneal fibroblasts of type 2 donors (Figure 2C). Anti-VEGFR-2 was negative (Figure 2B,D).

### Induction of corneal fibroblast network by VEGF

**Figure 3:**
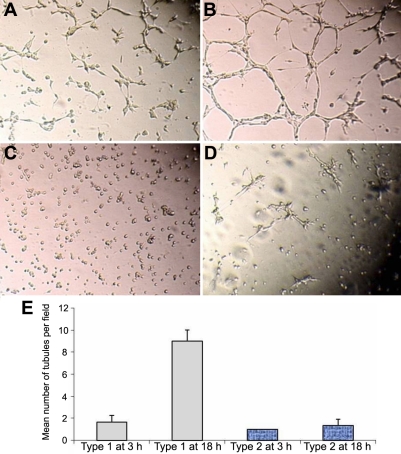
Figure-3 Induction of corneal fibroblast network by VEGF**. **The addition of exogenous VEGF (100 ng/m**l**), significantly increased the number of cell inter-connections in only type 1 cells after 3 h (**A**) and after 18 h (**B**). Type 2 fibroblasts do not respond to exogenous VEGF after 3 h (**C**) and after 18 h (**D**).  The number of tubules formed on Matrigel counted after at 3****h and 18****h and are presented in E.

To ascertain the role of VEGF receptors in the corneal fibroblast network formation, two groups of cells, type 1 and type 2, were studied. Figure 3 shows that the addition of exogenous VEGF (100 ng/ml) significantly increased the number of cell interconnections in only type 1 cells (Figure 3A,B). These results also indicate that type 2 fibroblasts do not respond to exogenous VEGF (Figure 3C,D). The number of tubules formed on Matrigel™ at 3 h and 18 h is presented in Figure 3E.

### Induction of corneal fibroblast migration by VEGF-A and VEGF-C

**Figure 4:**
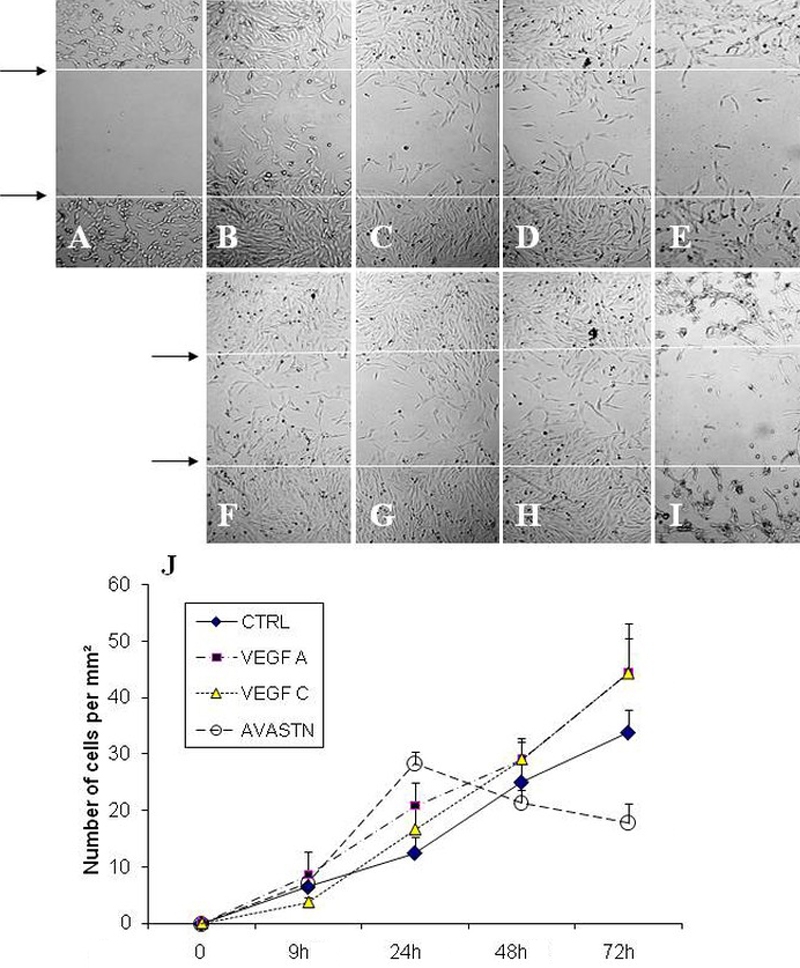
Corneal fibroblast migration induced by VEGF-A and VEGF-C as demonstrated by a wound healing assay: Upper panel:  **A** shows a photomicrograph of freshly created scar which is indicated as a space between****the two arrows**,** which serves as a reference for observing cell migration.  **B**, **C**, **D,** and **E** are photomicrographs after 24****h incubation while **F**, **G**, **H,** and **I** are photomicrographs taken after 72 h of incubation. **B** and **F** are untreated samples which serve as controls. **C** and **G** were treated with VEGF-A, while **D** and **H** were treated with VEGF-C. **E** and **I** were plates exposed to Avastin.  The number of cells migrating into the scar regions were counted in all samples at time 0, 9****h, 18****h, 24****h, 48****h**,** and 72****h of incubation and graph **J** was plotted.

As shown in Figure 4, cell motility and adhesion were counted using the “wound healing assay” at 9 h, 18 h, 24 h, 48 h, and 72 h (arrows indicate the site of freshly created scars). Figure 4A-E are micrographs after 24 h incubation while Figure 4F-I are pictures taken after 72 h of incubation. Figure 4B,F are untreated samples which serve as controls. Exposure of culture cells to VEGF-A (Figure 4C,G) and  VEGF-C (Figure 4D,H) significantly increased cell migration . However, cell migration was adversely affected when an anti-VEGF, Avastin, was added to the culture medium (Figure 4E,I). Avastin-mediated inhibition clearly suggests that the VEGFs play an important role in corneal fibroblast cell motility. The quantitative analysis of the cell number in different conditions is presented in Figure 4. Quantitative analysis as presented in the graph (Figure 4J) clearly confirms the microscopic observations. The effect of Avastin was noticeable already at 24 h, but at 72 h, cell migration suffered markedly in its presence when compared to controls. In the presence of VEGF-A and VEGF-B, the cell number at 72 h increased 1.5 times as compared to the control cultures, but it decreased 0.5 times instead with Avastin.

### Behavioral properties of cells transfected with VEGFR siRNA

Immunofluorescence studies presented in Figure 2 indicate that VEGFR-1 proteins are present in the human corneal fibroblast type 1 whereas it is undetectable in type 2. However, both types are negative for the presence of VEFGR-2.

**Figure 5:**
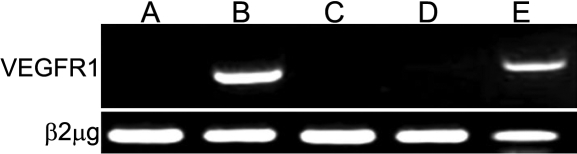
*VEGFR1* mRNAs detection in fibroblasts of type 1 and type 2 by RT-PCR. Slots B and E show that type 1 fibroblasts express *VEGFR1* mRNA whereas type 2 cells do not (slots A, C, and D).  β2-microglobulin mRNA served as an internal control.

We have investigated the presence of VEGFR-1 mRNAs in fibroblasts type 1 and type 2 by RT–PCR analysis. Type 1 fibroblasts express *VEGFR-1* mRNA (Figure 5, slots B and E) whereas type 2 do not (Figure 5, slots A, C, and D). We also transfected *VEGFR-1* specific siRNA into type-1 fibroblasts, using as controls experience negative *VEGFR-2* and *VEGFR-3* siRNAs or a scrambled siRNA provided by Qiagen.

**Figure 6:**
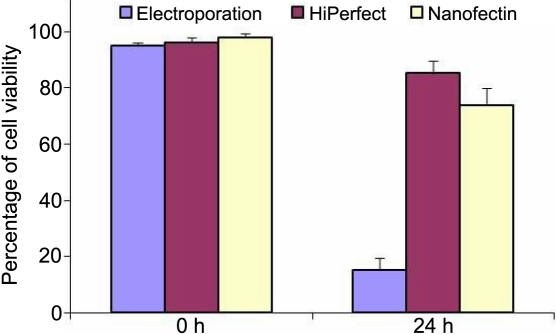
Cell viability for each of the three methods: electroporation, Hiperfect, and Nanofectin. Determination of cell viability at 0 h and after 24 h for siRNA transfection experiments using electroporation, Hiperfect, and Nanofectin. The result was plotted as a histogram.

We compared three methods of cell transfection, namely using: i) Amaxa electroporation, ii) Hiperfect® reagent, and  iii) Nanofectin®. Cell viability by trypan blue exclusion was performed at time 0 h and found to be above 95%. We observed that viability was remarkably different for the 3 different methods tested 24 h after transfection (15% of cell viability with Amaxa electroporation, 85% with HiPerfect®, and 81% with Nanofectin®).The results are presented in Figure 6. We have used the Hiperfect® method for further experiments because it gave us the best results.

**Figure 7:**
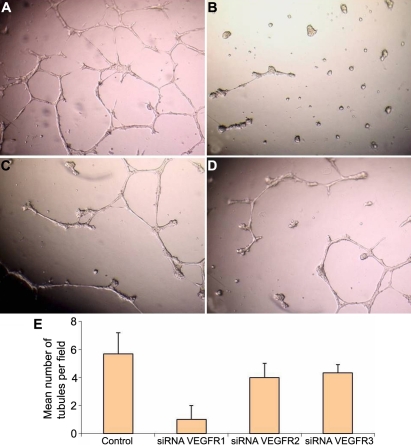
The plasticity of type 1 fibroblasts transfected with *VEGFR1*,*VEGFR2*, and *VEGFR3* siRNAs as demonstrated on Matrigel after 18 h of incubation. **A**: Non transfected corneal fibroblasts; **B**: transfected with *VEGFR1* siRNA; **C**: transfected with *VEGFR2* siRNA; and **D**: transfected with *VEGFR3* siRNA.  The number of tubules per field was counted and the mean was plotted as a histogram (**E**).

The plasticity of transfected type 1 fibroblasts was analyzed on Matrigel™ in parallel with control cells. Untreated corneal cells (control) readily organized into networks when grown on Matrigel™ substratum (Figure 7A). Transfection with *VEGFR-2* and *VEGFR-3* siRNAs or scrambled siRNA (results not shown), which were used as controls as indicated above, gave results comparable to the untreated control. In contrast, cell transfection by *VEGFR-1* siRNA (Figure 7B) dramatically decreased cell motility and network formation whereas *VEGFR-2* siRNA (Figure 7C) and *VEGFR-3* siRNA (Figure 7D) did not significantly affect the network formation.

In addition, the number of tubules formed by corneal fibroblast cells transfected with the three different siRNAs (*VEGFR-1*, *VEGFR-2*, and *VEGFR-3*) after 18 h of incubation is presented in Figure 7E. These results confirm again that *VEGFR-1* inhibition (and to a much lesser extent *VEGFR-2* and *VEGFR-3* inhibition) adversely affects tubule forming properties of corneal fibroblast type 1.

Our results show that the down-regulation of VEGFR-1 synthesis also adversely affects cell motility and network formation. Our data, therefore, suggests that VEGFR-1 is an important actor in the functional organization of corneal stromal fibroblasts.

### Age dependent decrease of VEGFR-1 expression

**Figure 8:**
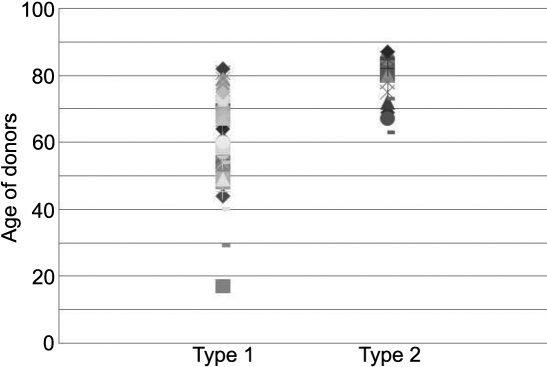
Distribution of Type 1 and Type 2 fibroblasts as a function of age of donors. Compilation of information yielded from experiments on cell reticulation on Matrigel and the presence or absence of VEGFR1 allowed segregation of donors in the two categories,  type 1 and type 2. It was noticed from donor data that type 1 was present in all age groups while type 2 was absent in young individuals (below 60 years). Type 2 was the predominant cell population in aged individuals.

Statistical analysis showed that the capacity of corneal fibroblasts to form networks on Matrigel™ is directly related to the age of the donors (Figure 8). Type 1 fibroblast cells predominated the corneal stroma in young donors whereas with increasing age, type 2 fibroblast cells begin to take over. In fact, the 70-90-year-old age group was composed of donors for type 2 fibroblast cells. Our results indicate that the expression of VEGFR-1 decreases with age.

## Discussion

Cell reticulation of stromal fibroblasts is a fundamental requisite for lamellar organization in cornea formation. Events that hinder this physiologic process can have heavy consequences. One sees one’s surroundings through the corneal structure without even being aware that one is looking through a well organized and structured organ. Its architecture and organization are primordial to its physiologic functions. Large amounts of data have been generated to understand corneal function in diseases and aging. However, much still remains to be understood. For the last few years, we have been focusing our research interest in understanding human corneal functions and notably ECM-associated corneal disorders. In a recently published work [1], we have provided evidence regarding the role of the fibulin family proteins in the organization of supramolecular ECM structures of the cornea. Another major finding that we reported concerns the production of protease type plasminogen activator (t-PA) and their inhibitors (PAI) in the human cornea and the alteration of these secretions (t-PA and PAI) in Schnyder's corneal dystrophy [26,27].

In the present work, we have attempted to further our current knowledge on the implication of growth factors in stromal networking and organization of the cornea. Our results indicate that some of the corneal fibroblasts can form cell-cell reticulation in vitro, which may be an indication of what is happening in vivo. We can distinguish two cell types (type 1 and type 2). Type 1 cells are the most abundant in individuals up to nearly sixty years of age, whereas type 2 cells make their appearance with increasing age when physiological body functions are affected and vision begins to diminish.

Cell type 1 properties and functions are intimately related to the expression of growth factor receptor, VEGFR-1. Exposure to antagonist (Avastin) and siRNA abolishes the capacity of these fibroblasts to migrate, establish interdendritic contacts, and organize into a functional reticulum during stromal lamellae formation. The proliferative and migratory effect as well as tubule and junction formation can be enhanced considerably by exogenous addition of VEGFR-1 to in vitro culture cells in our Matrigel™ system. VEGFR-1 therefore has a preeminent role in fibroblast cell migration, interdendritic contact formation, and the functional organization of the reticulum in stromal lamellae. These constitute a cascade of interrelated events leading to the formation of a healthy and functional cornea.

VEGFR-1 is a high affinity receptor for VEGF-A, VEGF-B, and PlGF. On the other hand, VEGFR-2 is expressed in neither type 1 or 2 cells and therefore is perhaps of no apparent consequence in the structural and functional organization of the cornea.

VEGFR-1 is also expressed in vascular endothelial cells and non-endothelial cells including macrophages and monocytes [28], mesenchymal cells [29], and hematopoietic stem cells [30]. VEGFR-1 signaling is involved in the migration of monocytes/macrophages [31], and it has also been shown that VEGFR-1 plays a role in PlGF-mediated recruitment of hematopoietic stem cells (HSCs) from a quiescent state to a proliferative one in the bone marrow, favoring differentiation, mobilization, and reconstitution of hematopoiesis [32]. The addition of exogenous VEGF to cells seeded on Matrigel™ clearly affected cell motility and contact formation besides increasing cell proliferation. Our observation is in conformity and goes along with the results obtained with the hematopoietic cell system by other authors [25,28].

VEGFR-1 blocking antibodies have been shown to prevent migration but not proliferation of HUVEC (human vascular endothelial cells) in response to VEGF-A, thereby indicating the involvement of VEGFR-1 in endothelial cell migration [33]. It has also been suggested that VEGFR-1-mediated signaling preferentially modulate the reorganization of actin via p38 MAPK (Mitogen-activated protein kinases) whereas VEGFR-2 contributes to the reorganization of the cytoskeleton by phosphorylating FAK (focal adhesion kinase) and paxillin (a focal adhesion-associated adaptor protein) [34]. Our results from the gene array show that these molecules are constitutively expressed and may play an autocrine or paracrine role in maintaining hemostasis of corneal fibroblasts. Avastin, a humanized monoclonal VEGF antibody, blocks cell migration, which indicates, though indirectly, the autocrine function of constitutively expressed VEGFs.

The results of the “wound healing” study confirm the autocrine role of VEGFR-1 in cell type 1 in migration and tubule formation. The decrease in VEGFR-1 expression is related to the diminution of the autocrine function and probably a progressive loss of cell multiplication and cell renewal, which culminates in the appearances of type 2 cells and the gradual development of poor vision.

In conclusion, the corneal fibroblast reticulations as seen in vitro seem to be related to the level of VEGFR-1 expression, and this expression decreases with age. This may also reflect in vivo. Our observation is significant since in pathological conditions or in age-related vision, diminution manipulations of VEGFR-1 expression may provide a novel treatment approach. Restoring VEGFR-1 function/expression levels may hold promises in treating stromal corneal pathologies in the young as well as aging individuals. Development of an artificial cornea for the vision deprived is a hope that needs to be sustained through research initiatives. The present work is a preliminary contribution toward achieving this goal.
